# The Inhibitory Effects of Anacardic Acid on Hepatitis C Virus Life Cycle

**DOI:** 10.1371/journal.pone.0117514

**Published:** 2015-02-06

**Authors:** Jana Hundt, Zhubing Li, Qiang Liu

**Affiliations:** 1 VIDO-InterVac, University of Saskatchewan, Saskatoon, Saskatchewan, Canada; 2 VIDO-InterVac, Vaccinology and Immunotherapeutics Program, University of Saskatchewan, Saskatoon, Saskatchewan, Canada; 3 VIDO-InterVac, Vaccinology and Immunotherapeutics Program, Veterinary Microbiology, University of Saskatchewan, Saskatoon, Saskatchewan, Canada; Harvard Medical School, UNITED STATES

## Abstract

Hepatitis C virus (HCV) is a small positive-sense single-stranded RNA virus that causes severe liver diseases. Current anti-HCV therapies involving direct-acting antivirals have significantly enhanced efficacy in comparison to traditional interferon and ribavirin combination. However, further improvement is needed to eradicate HCV. Anacardic acid (AnA) is a phytochemical compound that can inhibit the activity of various cellular enzymes including histone acetyltransferases (HATs). In this study, we investigated the effects of AnA on different phases of HCV life cycle. Our data showed that AnA can inhibit HCV entry, replication, translation, and virion secretion in a dose-dependent manner with no measurable effects on cell viability. In addition, we showed that two HAT inhibitors and knocking down HAT (PCAF) by RNAi can reduce HCV replication, suggesting a mechanism of AnA’s inhibitory effects on HCV. Elucidation of the AnA-mediated inhibitory mechanism should facilitate the development of new drug candidates for HCV infection.

## Introduction

Hepatitis C virus (HCV), a member of the genus *Hepacivirus* within the virus family *Flaviviridae*, is able to establish chronic infection in humans, eventually leading to liver cirrhosis, hepatocellular carcinoma and liver failure [1,2]. More than 170 million people worldwide are infected with HCV, 80–85% of which are chronic infections. Annually, 3–4 million people are newly infected and up to 350,000 people die from HCV related liver diseases every year (World Health Organization, www.who.int). HCV vaccine development is hampered by viral genetic heterogeneity conferring immune evasion and the lack of relevant and practical animal models [1,3]. The standard treatment for hepatitis C uses pegylated interferon-α (PEG-IFN) combined with ribavirin. Dependent on HCV genotype, this treatment is successful in 45–80% of cases. It is very costly and often associated with strong side effects [4,5]. Recently, a few direct-acting antiviral (DAA) drugs targeting HCV nonstructural proteins have been licensed with improved efficacy. These DAAs have limitations, such as drug resistance, heavy pill load, high cost, and side effects [6–8]. Identification of host-targeting agents should help develop more efficient and pan-genotype antiviral therapies against HCV [6,9].

Anacardic acid (AnA) is a phytochemical mixture of 2-hydroxy-6-alkylbenzoic acid homologs first extracted from the cashew *Anacardium occidentale* [10,11]. AnA is one of the active components in plants used in traditional medicine all over the world, including *Gingko biloba* (Asia), *Amphipterygeum adstringens* (South America) and *Ozoroa insignis* (Africa) [12–14]. AnA has been demonstrated to have several potentially therapeutic activities such as anti-inflammatory, antimicrobial, antioxidant and antitumor activities [14–17].

In the present study, we evaluated the effects of AnA on the HCV life cycle. We demonstrated that AnA inhibits HCV entry, replication, translation, and virion secretion in a dose-dependent manner. Furthermore, inhibition of histone acetyltransferase activities by two chemical inhibitors and RNAi suppressed HCV replication, suggesting a possible mechanism by which AnA exerts its inhibitory effect on HCV.

## Material and Methods

### Plasmids, inhibitors and antibodies

HCV genomic plasmids HCV-2a J6/JFH-1 and HCV-2a J6/JFH-1(p7-rLuc2A) harboring *renilla* luciferase (rLuc) reporter were received from Dr. Charles Rice [18]. Lentivirus production plasmid psPAX2 was obtained from Dr. Didier Trono as Addgene Plasmid 12260. HCV RNA translation reporter plasmid pHCV16LUC and lentivirus vector based RNAi plasmid for p300/CBP-associated factor (PCAF) were kindly provided by Drs. Matthias Gromeier, Shelton Bradrick, and Qiwei Zhai, respectively [19,20]. pTRIP-CMV-Luc was produced by inserting the luciferase gene into a lentiviral vector plasmid pTRIP (Open Biosystems). pcDNA3.1(+)-HCV-2a J6 core-E1–E2 was generated by cloning HCV-2a J6 core-E1–E2 sequence into pcDNA3.1(+) vector (Invitrogen). Anacardic acid (AnA, 2-Hydroxy-6-pentadecylbenzoic acid, C_22_H_36_O_3_, Alexis Biochemicals, ENZO Life Sciences), histone acetyltransferase inhibitors p300i (4-(4-{[5-(4,5-dimethyl-2-nitrophenyl)furan-2-yl]methylidene}-3-methyl-5-oxo-4,5-dihydro-1H-pyrazol-1-yl)benzoic acid, Calbiochem) and HATIIi (2,6-*bis*-(3-Bromo-4-hydroxybenzylidene)cyclohexanone, Calbiochem) were prepared in DMSO and stored at -20°C. Antibodies against HCV NS5A protein and *β*-actin were purchased from Virogen and Cell Signaling Technology, respectively.

### Cell lines

HuH-7, HuH-7.5 and HEK293T cells were cultured in Dulbecco’s Modified Eagle Medium (DMEM, Sigma) with 10% (v/v) fetal bovine serum (FBS, Life Technologies) at 37°C and 5% CO_2_. HuH-7-HCV-2a J6/JFH-1 and HuH-7-HCV-2a J6/JFH-1(p7-rLuc2A) genomic replicon cells were generated by stably transfecting HuH-7 cells with *in vitro* transcribed RNA under the selection with G418 (ENZO Life Sciences).

### *In vitro* transcription, RNA transfection, RNA and protein extraction

HCV-2a J6/JFH-1(p7-rLuc2A), HuH-7-HCV-2a J6/JFH-1 and HCV translation reporter RNA was produced by *in vitro* transcription from linearized plasmid DNA using the MEGAscript T7 kit (Life Technologies). For RNA transfection, cells were plated at 2.5x10^5^ density per well in 6-well plates and cultured overnight. The cells were transfected with 3 μg RNA using JetPEI reagent (Polyplus) and incubated for 4 h. Then the media were replaced with DMEM/1% FBS containing inhibitors at indicated concentrations or DMSO as control. Culture media was replaced every 24 h. At the end of the experiments, cells were washed with PBS and lysed in 1 mL TRIzol (Life Technologies) for 5 minutes at room temperature. RNA and protein were isolated according to the manufacturer’s protocols. The protein pellet was dissolved in 100 μL Laemmli buffer (60 mM Tris-HCl pH 6.8, 2% SDS, 10% glycerol, 0.5% *β*-mercaptoethanol, 0.01% bromophenol blue).

### Reverse transcription and real-time PCR (RT-PCR)

RNA extracted from cells with TRIzol (Life Technologies) was reverse transcribed into cDNA by Superscript II (Invitrogen) as previously described [21]. Real-time PCR experiments were performed with primers PCAF-FD (5′ GCCCTAGCTGCTCATGTTTC 3′) and PCAF-rev (5′ GGTTTTTCAAATGGGGGTTT 3′) using SYBR green based detection system. The transcript level of β-glucuronidase (GUSB) was determined in parallel with primers GUSB-FD (5′ GGTGCTGAGGATTGGCAGTG 3′) and GUSB-rev (5′ CGCACTTCCAACTTGAACAGG 3′) and used for normalization.

### Cell viability MTT assay

The cytotoxic effects of AnA on HuH-7-HCV-2a J6/JFH-1(p7-rLuc2A) cells were determined by the MTT assay. Briefly, cells in 96-well plates were incubated with different concentrations of AnA. Equal amounts of DMSO were used as solvent control. At different time points, cells were washed with PBS and incubated with 0.5 mg/mL MTT (diluted in 1% FBS containing media) for 4 h at 37°C. The MTT reagent was removed and 200 μL of DMSO added to each well to solubilize formazan. After gentle shaking for 5 min, optical density was measured at 560 nm. Background absorbance at 670 nm was subtracted from absorbance at 560 nm. Absorbance_560–670nm_ of DMSO treated cells were set to 100%.

### Luciferase reporter assay

At predetermined time points, cells were washed with PBS and harvested in Passive Lysis Buffer (Promega), and firefly luciferase or *renilla* luciferase activities were measured with Luciferase Assay Systems (Promega). Luciferase activity was normalized to protein concentration quantified by Bradford assay according to the manufacturer’s protocol (Bio-Rad).

### Western blot

Protein samples in Laemmli buffer were subjected to SDS-PAGE and transferred to nitrocellulose membranes. The blots were blocked in 5% skim milk in PBS for 1 h and incubated with primary antibody (1:1000 in PBS/0.1% Tween-20) overnight at 4°C. After washing with PBS, the blots were incubated with an infrared dye-labeled secondary antibody (1:10000, Li-Cor Biosciences) for 1 h at room temperature and then washed again. The blot was scanned using an Odyssey Infrared Imaging System (Li-Cor Biosciences) and fluorescent intensity of protein bands was quantified using the Odyssey software.

### HCV pseudoparticle (HCVpp) entry assay

HCV lentiviral pseudoparticles carrying luciferase reporter gene (HCVpp-Luc) were produced in HEK293T cells by co-transfecting psPAX2, pTRIP-CMV-Luc and pcDNA3.1(+)-HCV-2a J6 core-E1–E2 using the calcium phosphate precipitation method [22]. The supernatant containing the pseudoparticles was collected 48 h after transfection and briefly centrifuged before use. HuH-7.5 cells were pretreated with inhibitors at indicated concentrations or DMSO for 12 h, and then infected with HCVpp-Luc. Medium was replaced with medium 4 h post-infection. Luciferase reporter assay was performed 48 h post-infection.

### HCV infectious virion secretion assay

HuH-7-HCV-2a J6/JFH-1(p7-rLuc2A) replicon cells were treated with inhibitors at indicated concentrations or DMSO for 5 h after washed with phosphate buffered saline (PBS) to remove secreted virions. The inhibitors in the supernatant were removed by buffer exchange using Amicon Ultra-15 Centrifugal Filter units (Millipore). HuH-7.5 cells were infected with the supernatant for 4 h followed by replacement with fresh medium. Luciferase reporter assay was performed 72 h post-infection.

### Statistical analysis

All experiments were performed for three times and statistical differences were analyzed by Student’s *t* test. A *p* value of ≤ 0.05 was considered statistically significant.

## Results

### Anacardic acid inhibits HCV replication and translation

**Fig 1:**
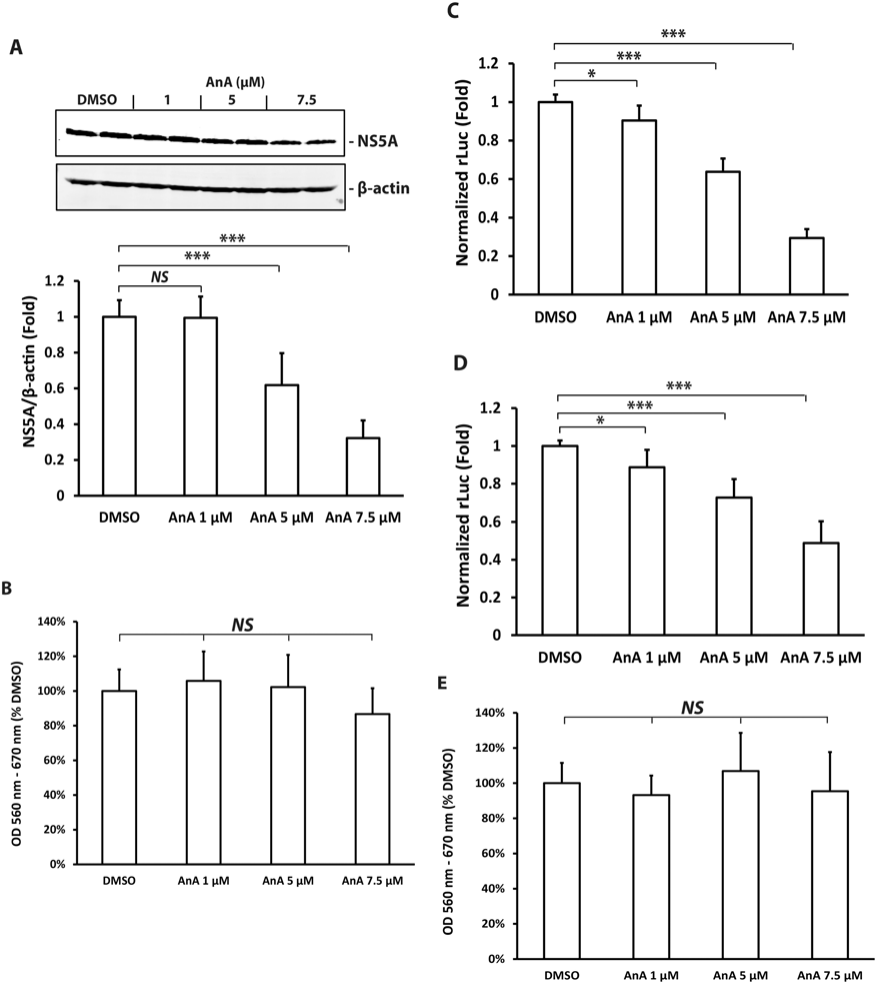
Anacardic acid (AnA) inhibits HCV replication and translation. (**A)**. HuH-7-HCV-2a J6/JFH-1 replicon cells were treated with DMSO or different concentrations of AnA for 72 h. Cell lysates were prepared in Laemmli buffer and subjected to SDS-PAGE followed by Western blotting with anti-NS5A antibody. The blot was shown in duplicates. NS5A expression was normalized to *β*-actin levels. NS5A/*β*-actin ratios were analyzed by Student’s *t* test and statistical significance is indicated as *** if *p* ≤ 0.001. (**B)**. Viability of cells treated as in (**A)** was determined by MTT. *NS* = not significant. **(C)**. HuH-7-HCV-2a J6/JFH-1(p7-rLuc2A) replicon cells were treated with DMSO or different concentrations of AnA for 48 h. *Renilla* luciferase activity was measured and normalized to total protein amount. Statistical differences between samples are indicated as * if *p* ≤ 0.05, or *** if *p* ≤ 0.001. **(D)**. HuH-7 cells were pre-treated with different concentrations of AnA for 48 h and transfected with HCV translation luciferase reporter RNA. Luciferase assay was performed 8 h after RNA transfection and normalized to total protein amount. Statistical differences between samples are indicated as * if *p* ≤ 0.05, or *** if *p* ≤ 0.001. **(E)**. Viability of cells treated as in (**C and D)** was determined by MTT. *NS* = not significant.

Given the multiple activities of AnA identified previously, we investigated whether AnA could modulate distinct components of the HCV life cycle. HCV-2a J6/JFH-1 genomic replicon cells were treated with different concentrations of AnA for 48 or 72 h and expression of viral protein (NS5A) levels were determined by Western blotting. After 48 h incubation with AnA, we did not observe significant changes in HCV protein levels (data not shown). Extended treatment with AnA to 72 h, however, decreased HCV NS5A protein levels in a dose-dependent manner as shown in Fig. 1A. AnA at 1 μM resulted in no detectable change, whereas AnA at 5 μM reduced NS5A levels to about 60% of that after DMSO treatment. AnA at 7.5 μM led to 70% reduction in NS5A levels (Fig. 1A). To validate this result, we used HCV-2a J6/JFH-1(p7-rLuc2A) reporter replicon cells where the level of *renilla* luciferase (rLuc) serves as a sensitive and rapid reporter for the degree of HCV RNA replication [18,23]. After AnA treatment for 48 h, AnA at 1, 5, and 7.5 μM resulted in 10, 40, and 70% reduction respectively in rLuc levels in comparison to DMSO treatment (Fig. 1C). The faster kinetics observed in rLuc reporter replicon cells (48 h) as measured by the luciferase assay when compared with NS5A level determination by Western blotting is presumably due to different sensitivities associated with the detection methods. To investigate the effects of AnA treatment on the viability of HCV-2a J6/JFH-1(p7-rLuc2A) replicon cells, we performed an MTT assay. AnA treatment at 1, 5, and 7.5 μM for 72 h (Fig. 1B) or 48 h (Fig. 1E) did not have measurable effects on cell viability. These results demonstrate that AnA inhibits HCV RNA replication in a dose-dependent manner.

To determine the effect of AnA on HCV RNA translation, we used an HCV translation reporter pHCV16Luc where the rLuc was cloned between HCV 5′ and 3′ un-translated regions and the rLuc level reflects the degree of HCV RNA translation [19]. HuH-7 cells were pre-treated with different concentrations of AnA for 48 h prior to transfection with HCV translation reporter RNA. Measuement of luciferase 8 h after RNA transfection indicated that AnA at 1, 5, and 7.5 μM could inhibit HCV RNA translation by approximately 10, 30, and 50% respectively compared to DMSO treatment (Fig. 1D). Taken together, these results indicate that AnA can inhibit HCV RNA replication and translation in a dose-dependent manner.

### Inhibitory effect of anacardic acid on HCV entry and secretion

**Fig 2:**
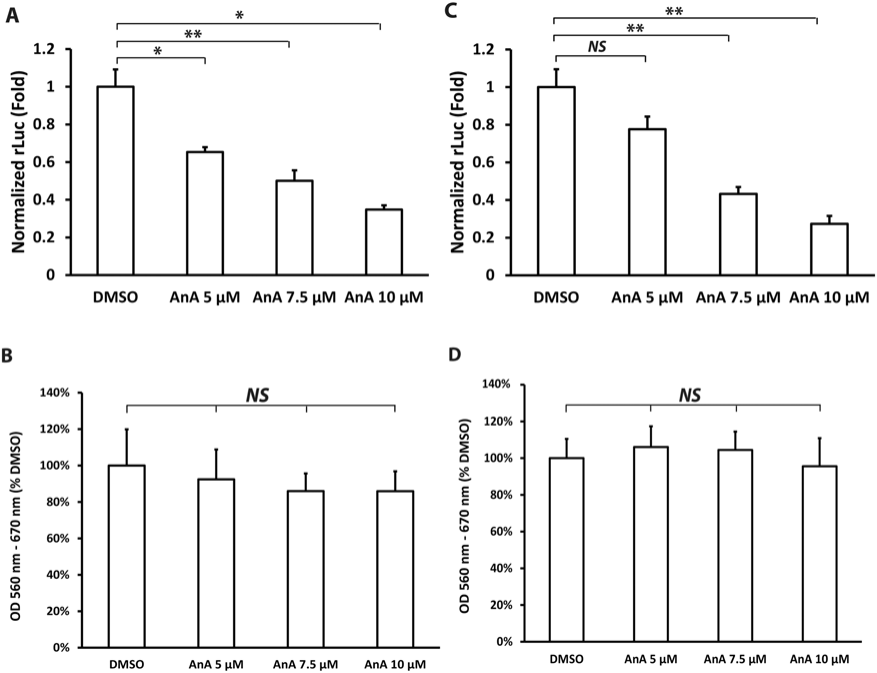
AnA inhibits HCV entry and release. (**A)**. HuH-7.5 cells were pretreated with DMSO and different concentrations of AnA for 12 h and infected with HCVpp-Luc for 4 h. followed by replacement by fresh medium. Luciferase activity was measured 48 h after infection and normalized to total protein amount. Statistical differences between samples are indicated as * if *p* ≤ 0.05, and ** if *p* ≤ 0.01. (**B)**. Viability of cells treated as in (**A)** was determined by MTT. *NS* = not significant. **(C)**. HuH-7-HCV-2a J6/JFH-1(p7-rLuc2A) cells were treated with DMSO and different concentrations of AnA for 5 h. After removal of inhibitors in the supernatant, HuH-7.5 cells were infected with the supernatant for 4 h followed by replacement of fresh medium. *Renilla* luciferase activity was measured 72 h after infection and normalized to total protein amount. Statistical differences are indicated as ** if *p* ≤ 0.01, or *NS* for not significant. **(D)**. Viability of cells treated as in (**C)** was determined by MTT. *NS* = not significant.

To elucidate whether AnA affects other stages of HCV life cycle, we studied the impact of AnA on HCV entry and secretion. We used lentiviral pseudoparticles (HCVpp) as a model for HCV entry. These are infectious lentiviral particles with HCV glycoproteins E1 and E2 incorporated onto lentiviral core particles, which provide an effective means to study HCV entry [24,25]. To evaluate the effect of AnA on HCV entry, HuH-7.5 cells were pretreated with different concentrations of AnA for 12 h, followed by infection with HCVpp. As shown in Fig. 2A, AnA at 5, 7.5 and 10 μM down-regulated HCVpp entry to approximately 60, 50, and 35% in comparison to the DMSO treatment. Under the same conditions, AnA treatments did not affect cell viability as measured by the MTT assay (Fig. 2B). Please note that we increased AnA concentrations in this experiment because of the relatively mild effects of AnA at 1 μM on HCV replication and translation observed in Fig. 1.

To determine the effect of AnA on HCV virion secretion, HuH-7-HCV-2a J6/JFH-1(p7-rLuc2A) cells were treated with AnA at the indicated concentrations or DMSO for 5 h after washing with PBS to remove secreted virions. The short incubation time was used to limit the possible inhibition on HCV protein production. After removal of inhibitors in the supernatant using the Amicon Ultra-15 Centrifugal Filter units to minimize their effect on HCV infection, HuH-7.5 cells were infected with the supernatant for 4 h followed by replacement with fresh medium. AnA at concentrations of 5, 7.5 and 10 μM resulted in 20, 50 and 70% reduction in HCV secretion (Fig. 2C) but with no observable effects on cell viability (Fig. 2D).

Taken together, these results demonstrate that AnA inhibits HCV entry and secretion in a dose-dependent manner.

### Inhibitory effect of histone acetyltransferase (HAT) inhibitors and knockdown on HCV replication

**Fig 3:**
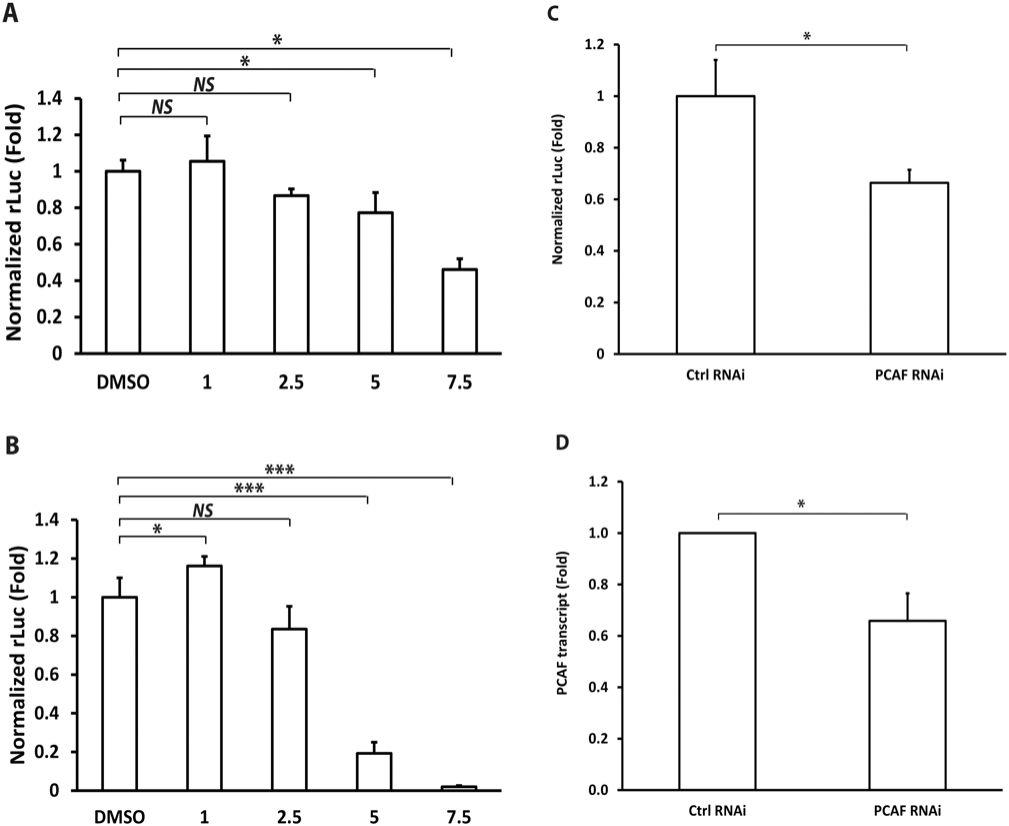
Histone acetyltransferase (HAT) inhibitors and knockdown suppress HCV replication. HuH-7-HCV-2a J6/JFH-1(p7-rLuc2A) replicon cells were incubated with DMSO or different concentrations of p300i (**A**) or HATIIi (**B**) for 48 h. *Renilla* luciferase activity was measured and normalized to total protein amount. Statistical differences are indicated as * if *p* ≤ 0.05, *** if *p* ≤ 0.001, or *NS* for not significant. **(C)**. HuH-7-HCV-2a J6/JFH-1(p7-rLuc2A) replicon cells were transfected with non-silencing control or PCAF RNAi. At 24 h after transfection, *renilla* luciferase activity was measured and normalized to total protein amount. Statistical differences are indicated as * if *p* ≤ 0.05. **(D)**. To determine the transcript level of PCAF, RNA was extracted from HuH-7-HCV-2a J6/JFH-1(p7-rLuc2A) replicon cells 16 h after transfecting with non-silencing control or PCAF RNAi. After reverse transcription, real-time PCR experiment was performed using PCAF-specific primers. The transcript level of β-glucuronidase (GUSB) was determined in parallel and used for normalization. Statistical difference is indicated as * if *p* ≤ 0.05.

One of the activities of AnA is inhibition of intracellular HATs including PCAF [26–28]. To examine whether the modulatory effect of AnA on HCV RNA replication is mediated through its HAT inhibitory function, we used two chemical HAT inhibitors p300i and HATIIi. We once again used HCV-2a J6/JFH-1(p7-rLuc2A) reporter replicon cells. As shown in Fig. 3A, treatment with p300i at 1 μM had no effect on HCV replication; 2.5, 5, and 7.5 μM p300i resulted in 10, 20, and 50% reduction in HCV replication. HATIIi at 1 μM resulted in a small increase in HCV replication whereas HATIIi at 2.5, 5, and 7.5 μM decreased HCV replication to 80, 20, and less than 5% in comparison to the DMSO treatment (Fig. 3B). The reason for the slight increase in HCV replication observed at 1 μM is unclear but previous studies demonstrated this concentration does not cause inhibition of HAT activities [29]. These data suggest that HAT inhibitors decrease HCV replication in a dose-dependent manner.

To substantiate the HAT inhibitor results, we knocked down the expression of a histone acetyltransferase PCAF by RNAi and determined the effect on HCV replication. For this purpose, HuH-7-HCV-2a J6/JFH-1(p7-rLuc2A) replicon cells were transfected with PCAF RNAi or a non-silencing control RNAi [20]. At 24 h after transfection, rLuc activity was determined by luciferase assay. As shown in Fig. 3C, transfection of PCAF RNAi decreased rLuc activity by more than 30% in comparison to control. PCAF knockdown by RNAi was confirmed by determining PCAF transcript level in RT-PCR experiment (Fig. 3D). These data suggest that knocking down PCAF decreases HCV replication.

Taken together, these results demonstrate that inhibition of HAT activities decreases HCV replication.

## Discussion

AnA has multiple pharmacological functions, such as anti-inflammatory, anti-cancer and antibacterial [26]. In this study, the effects of AnA on HCV life cycle were evaluated for the first time. Because there is no protocol for us to follow regarding the experimental conditions, we tested a few concentrations and treatment durations. We observed significant inhibitory effects on the HCV life cycle when AnA at ≥ 5 μM was used. As for the duration of the treatment, our results indicated that AnA inhibited HCVpp entry after 12 h treatment (Fig. 2A). A minimum of 48 h treatment with AnA was required before inhibition of HCV replication and translation were observed (Fig. 1). Treatment with AnA for only 5 h was sufficient to observe an effect on HCV virion production and release (Fig. 2C). The lack of synchrony between the inhibitory effects of different steps of the HCV life cycle by AnA suggests that inhibition is likely to occur through different mechanisms.

HCV entry is a tightly controlled process requiring numerous host factors including epidermal growth factor receptor (EGFR) [30]. Although AnA has not been shown to regulate EGFR signaling, AnA can inhibit vascular endothelial growth factor (VEGF) activity [31]. Given the close interaction and significant overlap between EGFR and VEGF signaling [32], future studies should investigate the involvement of EGFR signaling in HCV entry inhibition by AnA.

HCV RNA replication and translation are complex and can be modulated by both host and viral factors. It has been shown that AnA can inhibit histone acetyltransferase (HATs), lipoxygenase, and cyclooxygenase, as well as protein SUMOylation [26–28,33–35]. Using two HAT inhibitors and one HAT RNAi, we showed that HCV replication decreased when HATs were inhibited, suggesting a mechanism by which AnA may inhibit HCV replication. This finding is not unexpected since HCV core protein has been shown to activate host transcription by interacting with p300 [36]. In addition, recent studies showed that HCV infection activates lipogenic gene expression by p300-mediated transcription [37] and lipogenic gene expression is required for HCV replication [38,39].

However, it is very likely that other mechanisms may also play a role in the inhibitory effects of AnA on HCV replication. The previously reported inhibition of protein SUMOylation by AnA is another possible means by which AnA might perturb the HCV life cycle. SUMOylation is one type of post-translational modifications where a small ubiquitin-like modifier (SUMO) protein is covalently attached to a lysine residue in a target protein. SUMOylation is implicated in various cellular processes, including protein transport, protein stability, gene transcription, cell cycle regulation and chromatin organization [40–42]. Several viruses/viral proteins have been shown to exploit the host cell’s SUMOylation machinery, either by interacting with its components or by direct SUMOylation of viral proteins [43]. HCV NS5A protein was shown to be modified by SUMOylation and HCV replication was reduced by 30% when NS5A was not SUMOylated, suggesting a role of SUMOylation in HCV replication [44]. Future studies should be directed towards understanding whether inhibition of SUMOylation of cellular proteins by AnA has an effect on HCV life cycle.

The exact mechanisms behind HCV assembly and secretion are poorly understood. Data collected so far suggest that HCV assembly and secretion is closely linked to host very-low-density-lipoprotein (VLDL) and LDL pathway [30,45]. It is conceivable that AnA may interfere with this pathway.

In conclusion, we showed that AnA can inhibit HCV entry, replication, translation, and secretion in a dose-dependent manner with no measurable effects on cell viability. Our data suggest that at least part of the anti-HCV activity is through inhibition of HAT activities. Further elucidation of the molecular mechanism by which AnA inhibits different phases of HCV life cycle in future studies will provide attractive drug candidates for hepatitis C.
