# Correction: The Inhibitory Effects of Anacardic Acid on Hepatitis C Virus Life Cycle

**DOI:** 10.1371/journal.pone.0122379

**Published:** 2015-03-25

**Authors:** The PLOS ONE Staff

During typesetting, errors were introduced into the Funding section. The publisher apologizes for the error. The correct funding information is as follows: This work was supported by the Canadian Institutes of Health Research (RSN-109427) and the Saskatchewan Health Research Foundation to QL. ZL is a recipient of the University of Saskatchewan Vaccinology and Immunotherapeutics Ph.D. Student scholarship. The funders had no role in study design, data collection and analysis, decision to publish, or preparation of the manuscript.
